# LKB1-AMPK axis negatively regulates ferroptosis by inhibiting fatty acid synthesis

**DOI:** 10.1038/s41392-020-00297-2

**Published:** 2020-09-03

**Authors:** Changzhi Li, Xuan Dong, Wenjing Du, Xin Shi, Kangjie Chen, Wei Zhang, Minghui Gao

**Affiliations:** 1 The HIT Center for Life Sciences, School of Life Science and Technology, Harbin Institute of Technology, 150080 Harbin, China; 2 Department of Microbiology and Immunology, Weill Cornell Medicine 1300, York Avenue, New York, NY 10065 USA

**Keywords:** Cell biology, Cancer

**Dear Editor,**

Ferroptosis has emerged as a new programmed cell death modality highly relevant to disease.^1^ Ferroptosis was initially discovered as an iron-dependent non-apoptotic cell death induced by a serial of ferroptosis-inducing agents (FIN), such as erastin and RSL3. Erastin activates ferroptosis by inhibiting the activity of system Xc^−^, a cystine-glutamate antiporter, resulting in the depletion of cellular cysteine and glutathione (GSH), thus breaking cellular redox homeostasis. Inactivation of glutathione peroxidase 4 (GPX4), an enzyme required to remove the toxic lipid hydroperoxides can induce ferroptosis without the depletion of cellular cysteine and GSH.^1^

## AMPK negatively regulates ferroptosis

**Fig. 1:**
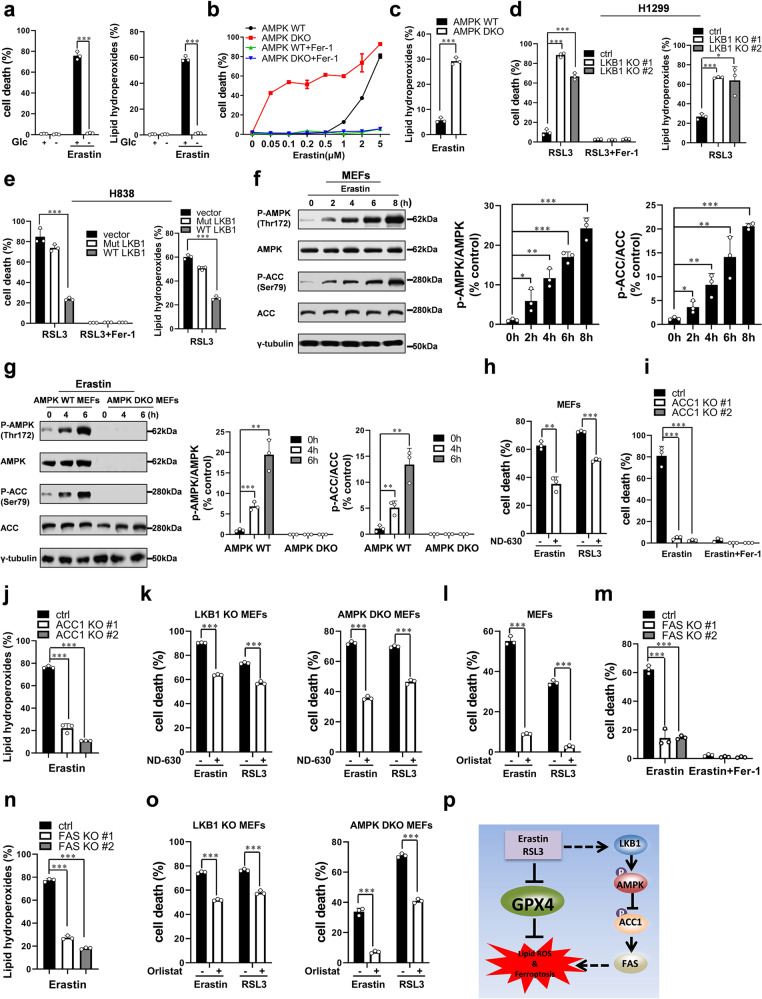
Negative regulation of ferroptosis by LKB1-AMPK axis mediated inhibition of fatty acid synthesis. **a** Glucose is essential for ferroptosis. MEFs were treated as indicated for 10 h. Cell death was determined by PI staining coupled with flow cytometry. For lipid hydroperoxides measurement, MEFs were treated as indicated for 8 h and lipid hydroperoxides was determined by BODIPY C11 staining coupled with flow cytometry. Glc Glucose; erastin, 10 μM. **b** AMPK DKO MEFs are more sensitive to ferroptosis than wild-type MEFs. Cells were treated by indicated ferroptosis inducers for 10 h for measurement of cell death. Cell death were measured by PI staining coupled with flow cytometry. Fer-1 ferrostatin-1, 2 μM. **c** Depletion of AMPK accelerates lipid hydroperoxides accumulation induced by erastin. Cells were treated with erastin (10 μM) for 8 h. **d** H1299 LKB1 KO cells are more sensitive to ferroptosis than wild-type cells. H1299 control and LKB1 KO cells were treated with RSL3 (1 μM) for 6 h to determine cell death. Cells were treated with RSL3 (1 μM) for 4 h to determine the accumulation of lipid hydroperoxides. **e** Overexpression of LKB1 sensitizes RSL3-induced ferroptosis and lipid hydroperoxidation in H838 cells. Left: H838 primed with WT (wide-type) or Mut (kinase dead mutant) LKB1were treated with RSL3 (25 μM) for 6 h, cell death was determined by PI staining coupled with flow cytometry. Right: H838 overexpressed with WT or Mut LKB1 were treated with RSL3 (25 μM) for 5 h, accumulation of lipid hydroperoxides was determined by BODIPY C11 staining coupled with flow cytometry. **f** Ferroptosis treatment activates AMPK in a time-dependent manner. Western blot analysis of phosphorylation of AMPK and ACC1 in MEFs treated with erastin for indicated time, erastin (1 μM). **g** AMPK is required for phosphorylation of ACC1 induced by erastin. Western blot analysis of phosphorylation of AMPK and ACC1 in wild-type MEFs and AMPK DKO MEFs treated with 1 μM erastin for indicated time. **h** Inhibitor of ACC1 can suppress erastin or RSL3-induced ferroptosis. MEFs were treated as indicated for 10 h and cell death was measured by PI staining. erastin (10 μM), RSL3 (0.5 μM), ND-630 (5 μM). **i**, **j** Knockout of ACC1 in MEFs blocks ferroptosis. Cells were treated as indicated for 10 h for measurement of cell death (**i**) or 8 h for measurement of lipid hydroperoxides (**j**), erastin (1 μM). **k** Inhibitor of ACC1 can suppress erastin or RSL3-induced ferroptosis in LKB1 KO MEFs and AMPK DKO MEFs. Cells were treated as indicated for 8 h and cell death was determined by PI staining coupled with flow cytometry. erastin (10 μM), RSL3 (1 μM), ND-630 (5 μM). **l** FAS inhibitor significantly inhibits ferroptosis in wild-type MEFs. MEFs were treated as indicated for 10 h, erastin (10 μM), RSL3 (0.5 μM), orlistat (50 μM). Cell death was determined by PI staining coupled with flow cytometry. **m**, **n** Lipid oxidation and ferroptosis require fatty acid synthesis. MEF control and FAS KO cells were treated as indicated for 10 h to determine cell death (**m**) or 8 h to determine lipid hydroperoxides (**n**), erastin (1 μM). **o** FAS inhibitor significantly inhibit ferroptosis in LKB1 KO MEFs and AMPK DKO MEFs. MEFs were treated as indicated for 10 h, erastin (10 μM), RSL3 (0.5 μM), orlistat (50 μM). Cell death was determined by PI staining coupled with flow cytometry. **p** Working model. LKB1-AMPK axis is activated by ferroptosis stimuli which in turn inhibits lipid synthesis to protect cells from ferroptosis. All quantitative data are presented as mean ± SD from three independent experiments. *P < 0.05, **P < 0.01, ***P < 0.001 by unpaired Student’s *t* test

In our previous study, glutamine was identified as a key extracellular regulator of ferroptosis. Glutaminolysis and functional TCA cycle and electron transport chain (ETC) are crucial for cysteine deprivation-induced ferroptosis.^2^ Glucose is another vital nutrition for ATP production. To test if glucose regulates ferroptosis, we treated mouse embryonic fibroblasts (MEFs) with erastin in glucose deprived medium. As shown in Fig. 1a, erastin failed to induce MEFs ferroptosis in a glucose-free medium. Lipid hydroperoxides are the hallmark of ferroptosis,^1^ we observed that erastin cannot induce lipid hydroperoxides accumulation in the absence of glucose (Fig. 1a), suggesting glucose is essential for cysteine deprivation-induced lipid hydroperoxides accumulation and ferroptosis. Similarly, glucose depletion reduced ferroptosis induced by RSL3, a highly specific GPX4 inhibitor (Supplementary Fig. 1a).

Since both glucose starvation and inhibiting the activity of TCA cycle and ETC decrease cellular ATP level and lead to the activation of AMPK,^3^ we investigated the involvement of AMPK in ferroptosis. Firstly, we observed that the pharmacological activator of AMPK, 5-aminoimidazole-4-carboxamide ribonucleotide (AICAR) remarkably blocked ferroptosis and associated lipid hydroperoxidation (Supplementary Fig. 1b, c). In contrast to this, double knockout (DKO) of AMPK α1 and α2 subunits significantly enhanced the sensitivity of MEFs to ferroptosis and accumulation of lipid hydroperoxides induced by erastin or RSL3 compared to wild-type MEFs (Fig. 1b, c, Supplementary Fig. 1d–h). And reconstituting wild-type but not the kinase dead mutant (K47R) AMPK α1 subunit back to AMPK DKO MEFs restored the ferroptosis sensitivity (Supplementary Fig. 1i–k). Using ferroptosis inhibitor ferrostatin-1 (Fer-1), we further confirmed that cells underwent ferroptosis (Fig. 1b, Supplementary Fig. 1e, h). NAD(P)H is a biomarker for sensitivity to ferroptosis inducers.^1^ Knockout of AMPK significantly sensitized cells to erastin induced NAD(P)H oxidation (Supplementary Fig. 1l). Ferroptosis is driven by inactivation of GPX4. Deletion of AMPK enhanced erastin induced GPXs inactivation (Supplementary Fig. 1m). We also found that mitochondria were impaired much worse in AMPK DKO MEFs than wild-type cells (Supplementary Fig. 1n).

Previous studies suggested that inhibition of AMPKα by compound C diminishes erastin-induced ferroptosis in cancer cells.^4^ However, we found compound C inhibited ferroptosis not only in wild-type MEFs but also in AMPK DKO MEFs, suggesting the inhibitory effect of compound C on ferroptosis is independent on AMPK (Supplementary Fig. 1o).

## Loss of function of the tumor suppressor LKB1 enhances sensitivity to ferroptosis in human non-small cell lung cancer cells

The main upstream kinase responsible for the activation of AMPK in response to energy stress is LKB1, which is one of the most commonly mutated tumor suppressors in several human cancers, particularly in non-small cell lung cancer cells (NSCLC) and cervical carcinomas.^3^

Considering our finding that AMPK negatively regulates ferroptosis, we next examined whether LKB1 is a negative regulator of ferroptosis using a panel of NSCLC cells. Ferroptosis was accelerated in two independent LKB1 KO H1299 clones (Supplementary Fig. 2a and Fig. 1d). And reconstituting wild-type but not the kinase dead mutant LKB1 (K78I) back to H1299 LKB1 KO cells can restore the ferroptosis sensitivity (Supplementary Fig. 2b–d). More importantly, we introduced LKB1 to H838, a LKB1-null NSCLC cell line and found wild-type but not kinase dead mutant LKB1 confers resistance to ferroptosis and lipid hydroperoxides accumulation in these cells (Fig. 1e and Supplementary Fig. 2e). Consistently, knocking down of LKB1 resulted H1975 and H358 cells significantly sensitive to ferroptosis (Supplementary Fig. 2f and Fig. 2g). Similarly, depletion of LKB1 sensitized MEFs to ferroptosis, lipid hydroperoxidation, and NAD(P)H oxidation (Supplementary Fig. 2h–m). Therefore, LKB1 is a negative regulator of ferroptosis and LKB1 status might be useful as a biomarker to predict non-small cell lung carcinoma responsiveness to the induction of ferroptosis.

## LKB1-AMPK negatively regulates ferroptosis by inhibitory phosphorylation of ACC1

Next, we sought to determine how AMPK regulates ferroptosis sensitivity. We found that ferroptosis stimuli induced remarkable phosphorylation of AMPK in both MEFs and H1299 cells (Fig. 1f and Supplementary Fig. 3a, b). Interestingly, ferrostatin-1 has no effect on FIN induced AMPK activation (Supplementary Fig. 3d, e). As shown above (Fig. 1b, c and Supplementary Fig. 1), knockout of AMPK sensitized cells to ferroptosis, suggesting the activation of AMPK might be a protective response upon FIN treatment.

Activation of AMPK results in cellular metabolism reprogramming.^3^ Given the curial role of lipid hydroperoxides in ferroptosis, we, therefore, focused on fatty acid synthesis. AMPK negatively regulates fatty acid synthesis by inhibitory phosphorylating ACC1, the rate-limiting enzyme for fatty acid biosynthesis, upon energy insufficiency stress.^3^ We observed ferroptosis stimuli induced robust phosphorylation of ACC1 in wild-type MEFs but not in AMPK DKO MEFs (Fig. 1f, g and Supplementary Fig. 3a, c), suggesting ACC1 is inhibitory phosphorylated by AMPK during ferroptosis. We subsequently investigated the role of ACC1 in ferroptosis. ND-630, a highly specific ACC1 inhibitor, significantly inhibited ferroptosis in wild-type MEFs (Fig. 1h). Consistently, ACC1 KO MEFs were remarkably resistant to ferroptosis, accumulated less lipid hydroperoxides and less NAD(P)H oxidation compared to wild-type MEFs (Fig. 1i–j and Supplementary Fig. 3f–j). Knockout of ACC1 protected cells from erastin induced mitochondrial damage and GPXs inhibition (Supplementary Fig. 3k, l). More importantly, ND-630 could partially inhibit ferroptosis in LKB1 KO MEFs and AMPK DKO MEFs (Fig. 1k). Consistently, knocking down of ACC1 in LKB1 KO MEFs significantly suppressed ferroptosis (Supplementary Fig. 3m, n). Taken together, these data suggest that ACC1 is partially required for the high sensitivity to ferroptosis in LKB1 and AMPK deficient cells.

## Fatty acid synthase is required for ferroptosis

Fatty acid synthase (FAS) catalyzes the de novo synthesis of fatty acids from simple precursors downstream of ACC1. We observed that ferroptosis were markedly blocked by Orlistat, a specific inhibitor of FAS in MEFs (Fig. 1l). Knockout of FAS significantly inhibited ferroptosis, lipid hydroperoxides, NAD(P)H oxidation and GPXs inhibtion in MEFs (Fig. 1m, n, Supplementary Fig. 4). Moreover, ferroptosis were markedly blocked by Orlistat in LKB1 KO MEFs and AMPK DKO MEFs (Fig. 1o). These results suggested ACC1-FAS mediated fatty acid biogenesis is required for ferroptosis, and inhibition of LKB1-AMPK promotes ferroptosis through the removal of inhibition of ACC1-FAS mediated fatty acid biogenesis.

Collectively, in this study we demonstrated that the LKB1-AMPK signal plays an important role in the regulation of ferroptosis. Upon induction of ferroptosis, AMPK is activated by upstream kinase LKB1, leading to the inhibition of cellular lipid synthesis by inhibitory phosphorylation of ACC1 and other probable substrates that are required for lipid bio-synthesis, which in turn protect cells from lipid hydroperoxides accumulation and ferroptosis (Fig. 1p). While we are preparing this manuscript, Lee et al also reported that AMPK negatively regulates ferroptosis by inhibiting fatty acid synthesis.^5^ As LKB1 is frequently mutated in cancer, this study has clear implications for cancer therapies. Our finding suggests that there might be a dose-responsive window for cancers that contain certain genetic signatures and that ferroptosis-inducing cancer therapies might have considerable benefits in overcoming cancer resistance to current treatments.

## Supplementary information

SUPPLEMENTAL MATERIAL AND FIGURE

## Data Availability

The data that support the findings of this study are available from the lead corresponding author (M.G.) on reasonable request.
