# Supplementary material for: LKB1-AMPK axis negatively regulates ferroptosis by inhibiting fatty acid synthesis
Source: Signal Transduct Target Ther. 2020 Sep 3;5:187. doi: 10.1038/s41392-020-00297-2 (PMC7471309; doi:10.1038/s41392-020-00297-2)
Supplement: Supplementary file 1 — SUPPLEMENTAL MATERIAL AND FIGURE [file 41392_2020_297_MOESM1_ESM.docx]

Supplementary materials for

LKB1-AMPK Axis Negatively Regulates Ferroptosis by Inhibiting Fatty Acid Synthesis

Changzhi Li^1^, Xuan Dong^1^, Wenjing Du^1^, Xin Shi^1^, Kangjie Chen^1^, Wei Zhang^2^* and Minghui Gao^1^*

*corresponding author: Wei Zhang: wez2009@med.cornell.edu

Minghui Gao: [gaominghui@hit.edu.cn](mailto:gaominghui@hit.edu.cn)

**Supplementary information, Figures**

Fig.S1 AMPK Negatively Regulates Ferroptosis.

Fig.S2 Loss of Function of LKB1 Enhances Sensitivity to Ferroptosis.

Fig.S3 LKB1-AMPK negatively regulates ferroptosis by inhibitory phosphorylation of ACC1

Fig.S4 Fatty Acid Synthase Is Required for Ferroptosis

**Supplementary information, Materials and Methods**


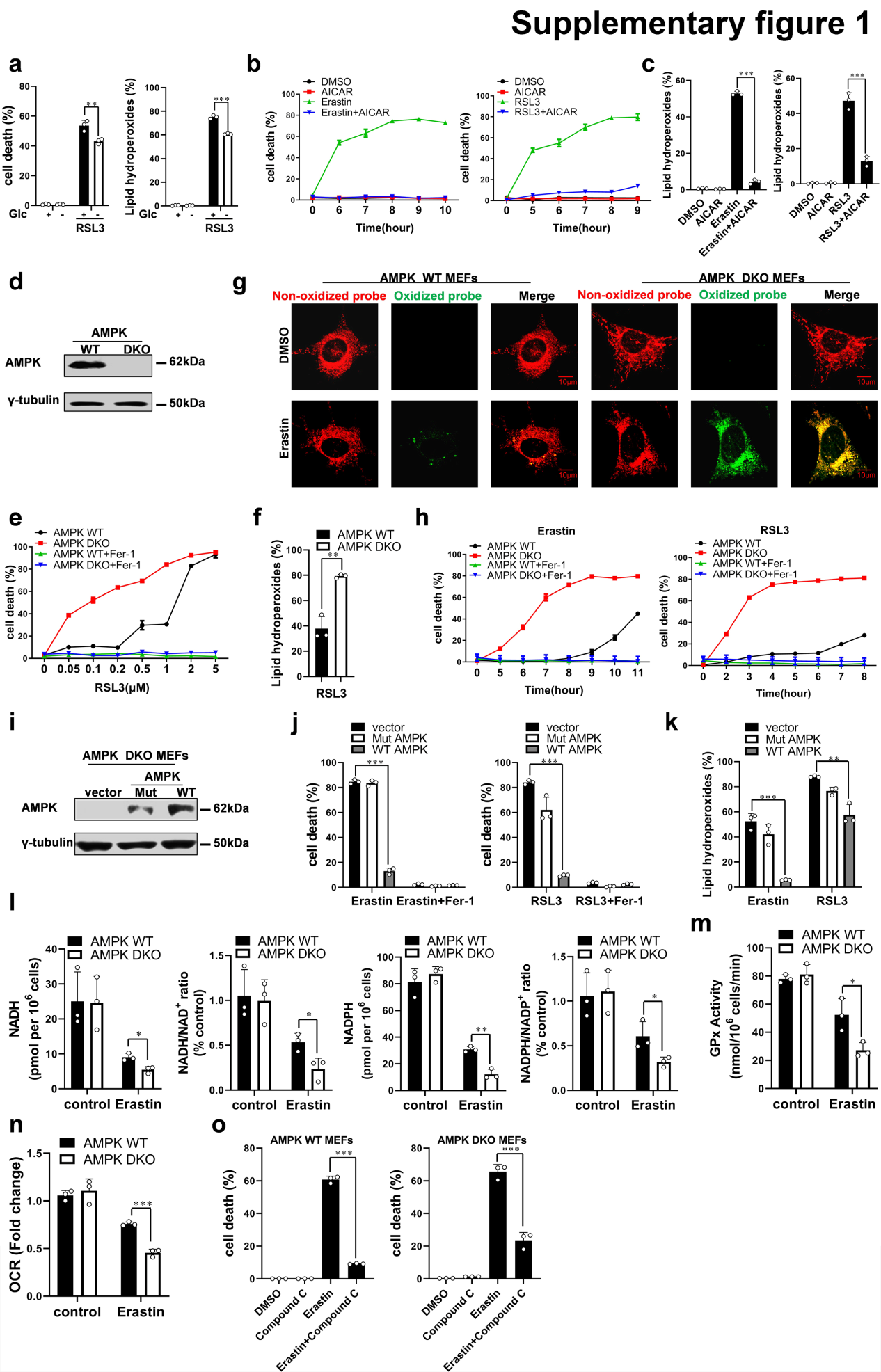


**Fig. S1** **AMPK Negatively Regulates Ferroptosis.**

(a) Glucose is essential for ferroptosis. MEFs were treated as indicated for 10 hr. Cell death was determined by PI staining coupled with flow cytometry. For lipid hydroperoxides measurement, MEFs were treated as indicated for 8 hr and lipid hydroperoxides was determined by BODIPY C11 staining coupled with flow cytometry. Glc, Glucose; RSL3, 1 μM. (b) AMPK activator AICAR blocks ferroptosis in MEFs. MEFs were treated as indicated and cell death was determined by PI staining coupled with flow cytometry. erastin (1μM) or RSL3 (0.5 μM). (c) AMPK activator AICAR inhibits lipid hydroperoxidation induced by ferroptosis stimuli in MEFs. MEFs were treated as indicated for 8 hr, and lipid hydroperoxides was determined by BODIPY C11 coupled with flow cytometry. erastin, 1 μM, RSL3, 0.5 μM. (d) Western blot confirming double knockout of AMPK ɑ1 and ɑ2 subunits in MEFs. (e) Depletion of AMPK sensitized cells to ferroptosis. Cells were treated by indicated ferroptosis inducers for 10 hr for measurement of cell death. Cell death were measured by PI staining coupled with flow cytometry. Fer-1: ferrostatin-1, 2 μM. (f) Depletion of AMPK accelerates lipid hydroperoxides accumulation induced by RSL3. Cells were treated with RSL3 (1 μM) for 8 hr. (g) Laser confocal microscopy detection of lipid hydroperoxides in AMPK WT MEFs and AMPK DKO MEFs. Cells were treated as indicated for 6 hr and then stained with BODIPY C11, erastin (10 μM). Oxidized probe (green) indicates lipid hydroperoxides. Scale bar, 10 μm. (h) Cell death were measured following treatment as indicated by PI staining coupled with flow cytometry. erastin (2 μM), RSL3 (1 μM), Fer-1: ferrostatin-1, 2 μM. (i-k) Reconstitution of AMPK DKO with wild-type (WT) but not kinase-dead mutant AMPK ɑ1 rescues ferroptosis sensitivity and lipid hydroperoxidation. (i) Western blot confirming reconstitution of AMPK DKO MEFs with vectors encoding WT or kinase-dead mutant (Mut) AMPK. (j and k) Cell Death were treated with erastin (10 μM) or RSL3 (0.5 μM) for 10 hr to determine cell death (j) or 8 hr to determine lipid hydroperoxides (k). (l) Depletion of AMPK promotes erastin induced NAD(P)H oxidation. Cellular NAD(P)H/NAD(P)^+^ was measured following treatment with erastin (10 μM) for 5 hr . (m) Knocking out of AMPK enhances erastin induced GPxs inactivation. GPXs activity was measured following treatment with erastin (10 μM) for 5 hr. (n) Knocking out of AMPK promotes erastin induced mitochondria damage. Mitochondrial oxygen consumption rate (OCR) was measured following treatment with erastin (10 μM) for 5 hr by Seahorse Cell Energy Phenotype Test Kit (Agilent Technologies) with Seahorse XFp Extracellular Flux Analyzer (Agilent Technologies). All quantitative data are presented as mean ± SD from three independent experiments. **P* <*0.05*, ***P* <*0.01*, ****P<0.001* by unpaired Student’s t test.


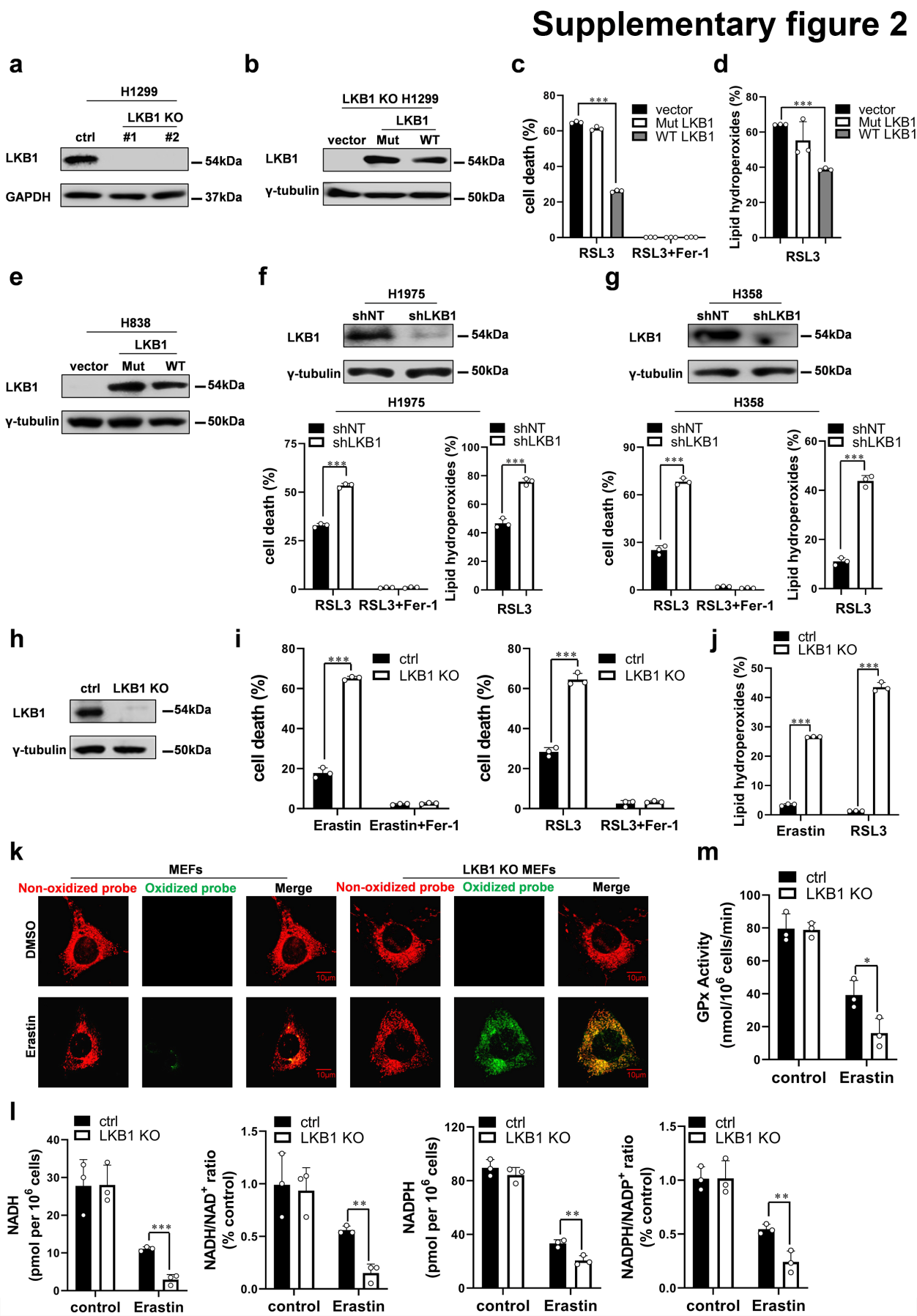


**Fig.S2 Loss of Function of LKB1 Enhances Sensitivity to Ferroptosis.**

(a) Western blot confirming knocking out of LKB1 in H1299 cells. (b-d) Reconstitution of LKB1 rescued ferroptosis sensitivity and accumulation of lipid hydroperoxides in NSCLC cells. (b) Western blot analysis of LKB1 expression in LKB1 KO H1299 cells reconstituted with vectors encoding wide-type (WT) or kinase-dead mutant (Mut) LKB1. (c and d) LKB1 KO H1299 cells reconstituted with WT or Mut LKB1 were treated with RSL3 (1 μM) for 6 hr to determine cell death (c) or 4 hr to determine lipid hydroperoxides (d). (e) Western blot analysis of the expression of LKB1 in H838 reconstituted with vectors encoding wide-type (WT) or kinase-dead mutant (Mut) LKB1. (f and g) Knockdown of LKB1 enhances ferroptosis sensitivity in NSCLC cells. Western blot confirming knocking down of LKB1 in H1975 or H358 cells. Cell death was measured following treatment with RSL3 (1 μM) for 6 hr. Lipid hydroperoxides was measured following treatment with RSL3 (1 μM) for 4 hr. (h-j) Knockout of LKB1 increases ferroptosis sensitivity in MEFs. (h) Western blot confirming knockout of LKB1 in MEFs. (i) Cells were treated with erastin (10 μM) or RSL3 (1 μM) for 10 hr, cell death was determined by PI staining coupled with flow cytometry. (j) Cells were treated as (i) for 8 hr, accumulation of lipid hydroperoxides was determined by BODIPY C11 coupled with flow cytometry. (k) Laser confocal microscopy detection of lipid hydroperoxides in MEF control and LKB1 KO cells. Cells were treated as indicated for 6 hr, and then stained with BODIPY C11, erastin (10 μM). Oxidized probe (green) indicates lipid hydroperoxides. Scale bar, 10 μm. (l) Depletion of LKB1 promoted erastin induced NAD(P)H oxidation. Cellular NAD(P)H/NAD(P)^+^ was measured following treatment with erastin (10 μM) for 5 hr . (m) Knocking out of LKB1 enhanced erastin induced GPxs inactivation. GPXs activity was measured following treatment with erastin (10 μM) for 5 hr. All quantitative data are presented as mean ± SD from three independent experiments. **P* <*0.05*, ***P* <*0.01*, ****P<0.001* by unpaired Student’s t test.


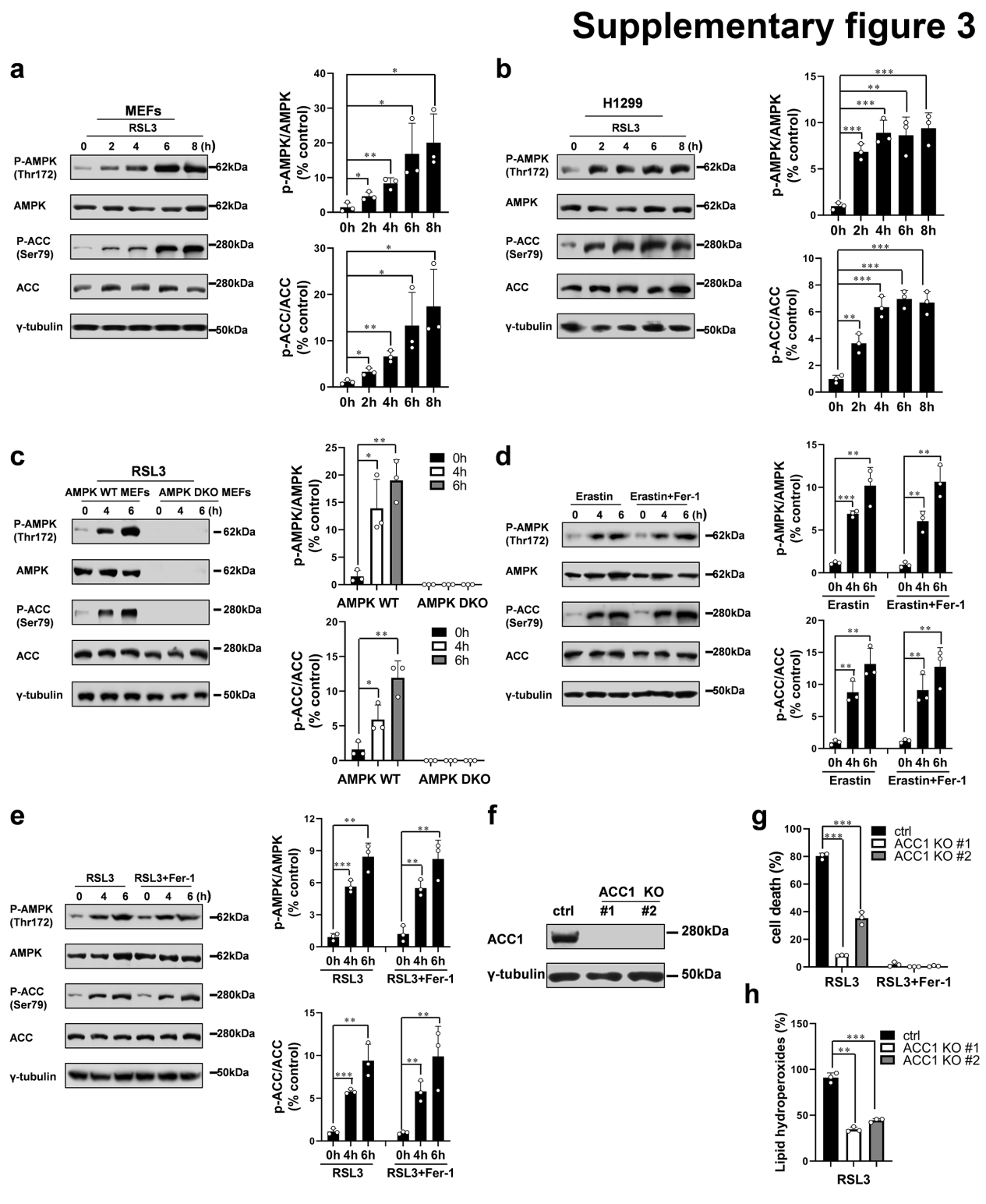


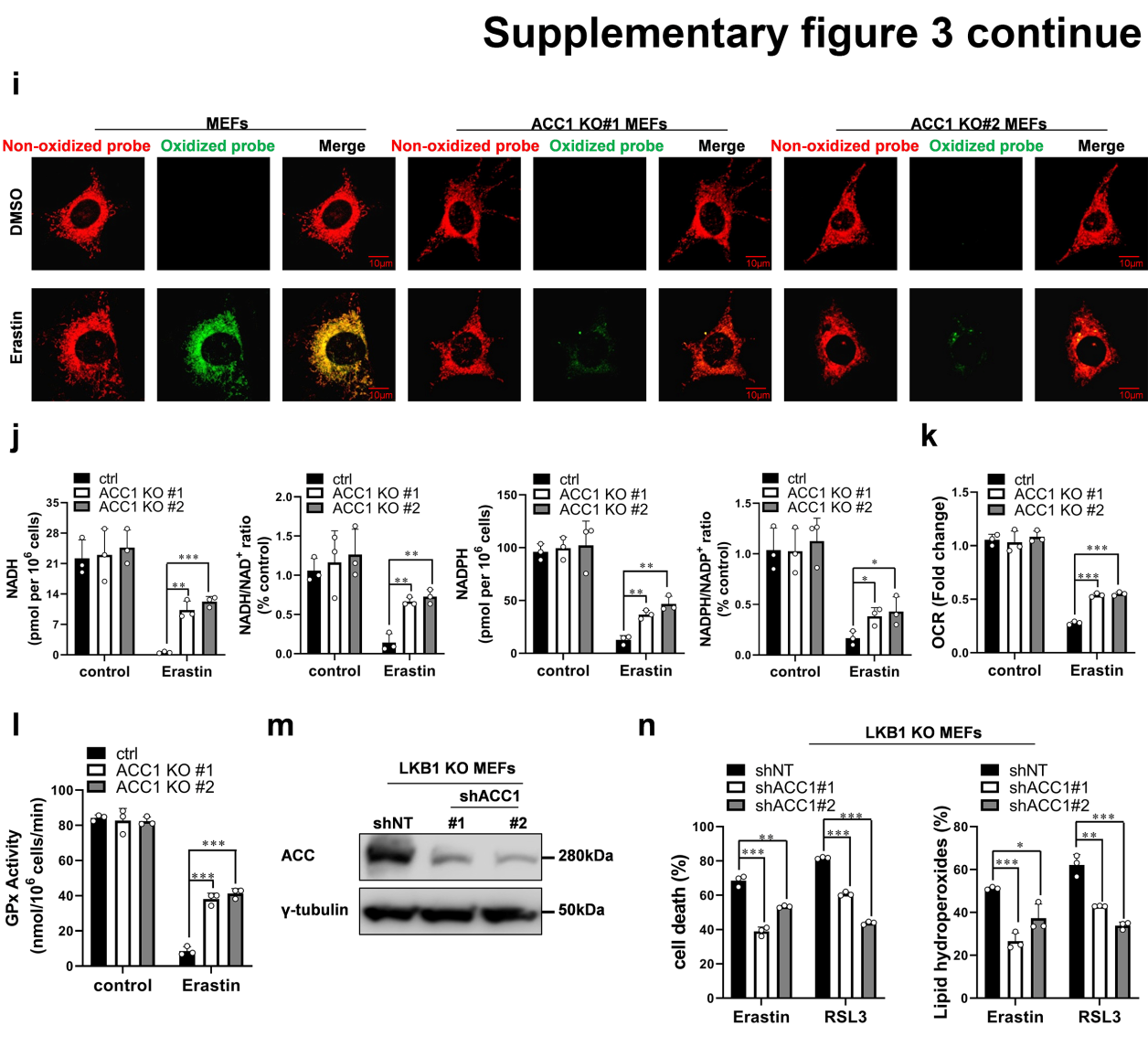
 **Fig.S3 LKB1-AMPK negatively regulates ferroptosis by inhibitory phosphorylation of ACC1**

(a-b) Ferroptosis treatment activates AMPK in a time-dependent manner. Western blot analysis of phosphorylation of AMPK and ACC1 in MEFs (a) or H1299 (b) treated with RSL3 for indicated time. 0.5 μM RSL3 for MEFs and 1μM RSL3 for H1299 . (c) AMPK is required for phosphorylation of ACC1 induced by ferroptosis stimuli. Western blot analysis of phosphorylation of AMPK and ACC1 in wild-type MEFs or AMPK DKO MEFs treated with 0.5 μM RSL3 for indicated time. (d and e) Ferrostatin-1 has no effect on FIN induced AMPK activation. Western blot analysis of phosphorylation of AMPK and ACC1 in MEFs treated with erastin (1 μM) (d) or RSL3 (0.5 μM) (e) with ferrostatin-1( Fer-1, 2 μM) for indicated time. (f) Western blot confirming knockout of ACC1 in MEFs. (g and h) Knockout of ACC1 in MEFs blocks ferroptosis. Cells were treated as indicated for 10 hr for measurement of cell death (g) or 8 hr for measurement of lipid hydroperoxides (h), RSL3 (0.5 μM). (i) Laser confocal microscopy detection of lipid hydroperoxides in MEF control and ACC1 KO cells. Cells were treated as indicated for 6 hr and then stained with BODIPY C11, erastin (1 μM). Oxidized probe (green) indicating lipid hydroperoxides. Scale bar, 10 μm. (j) Knocking out of ACC1 inhibits erastin induced NAD(P)H oxidation. Cellular NAD(P)H/NAD(P)^+^ was measured following treatment with erastin (1 μM) for 5 hr. (k) Knocking out of ACC1 suppresses erastin induced mitochondria damage. Mitochondrial oxygen consumption rate (OCR) was measured following treatment with erastin (10 μM) for 5 hr by Seahorse Cell Energy Phenotype Test Kit (Agilent Technologies) with Seahorse XFp Extracellular Flux Analyzer (Agilent Technologies) . (l) Depletion of ACC1 protects erastin induced GPxs inactivation. GPx activity was measured following treatment with erastin (1 μM) for 5 hr. (m and n) Knocking down of ACC1 can suppress erastin or RSL3-induced ferroptosis in LKB1 KO MEFs. (m) Western blot confirming knocking down of ACC1 in LKB1 KO MEFs. (n) Cells were treated with 10 μM erastin or 1 μM RSL3 for 10 hr to measure cell death (left) or 8 hr to measure lipid hydroperoxides (right). All quantitative data are presented as mean ± SD from three independent experiments. **P* <*0.05*, ***P* <*0.01*, ****P<0.001* by unpaired Student’s t test.


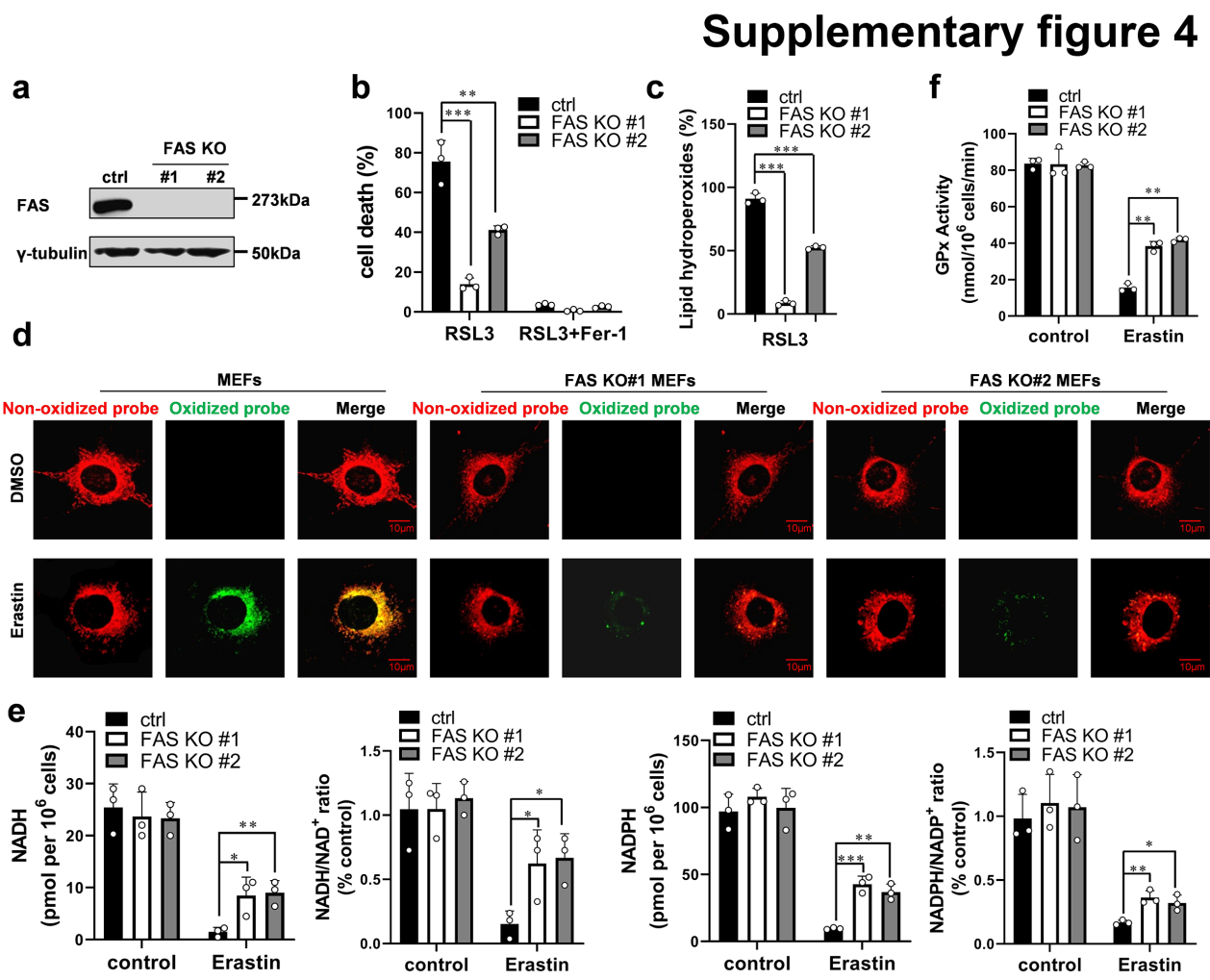


**Fig.S4 Fatty Acid Synthase Is Required for Ferroptosis**

(a) Western blot confirming knockout of FAS in MEFs. (b and c) FAS is required for RSL3 induced ferroptosis(b) and lipid hydroperoxidation(c). Cells were treated as indicated for 10 hr to determine cell death (b) or 8 hr to determine lipid hydroperoxides (c). RSL3 (0.5 μM). (d) Laser confocal microscopy detection of lipid hydroperoxides in MEF control and FAS KO cells. Cells were treated as indicated for 6 hr and then stained with BODIPY C11, erastin (10 μM). Oxidized probe (green) indicates lipid hydroperoxides. Scale bar, 10 μm. (e) Knocking out of FAS inhibits erastin induced NAD(P)H oxidation. Cellular NAD(P)H/NAD(P)^+^ was measured following treatment with erastin (1 μM) for 5 hr. (f) Depletion of FAS protects erastin induced GPxs inactivation. GPx activity was measured following treatment with erastin (1 μM) for 5 hr. All quantitative data are presented as mean ± SD from three independent experiments. **P* <*0.05*, ***P* <*0.01*, ****P<0.001* by unpaired Student’s t test.

**MATERIALS AND METHODS**

**Reagents and antibodies**

Primary antibodies used were anti-AMPK (CST, Cat# 5831S), anti-P-AMPK (CST, Cat# 2535S), anti-ACC (CST, Cat# 3662S), anti-P-ACC (CST, Cat# 3661S), anti-γ-tubulin (Sigma, Cat# T6557), anti-LKB1 (abcam, Cat# ab15095), anti-GAPDH (Santa Cruz, Cat# A1918), Erastin (Selleck, Cat# S7242), RSL3 (Selleck, Cat# S8155), AICAR(Selleck, Cat# S1802) , Compound C (Selleck, Cat# S7306), Ferrostatin-1 (Fer-1) (Selleck, Cat# S7243), ND-630 (MCE, Cat# HY-16901), Orlistat(Selleck, Cat# S1629).

**Cell culture**

Unless specified otherwise, all mammalian cells are maintained in DMEM with high glucose, sodium pyruvate (1 mM), glutamine (4 mM), penicillin (100 U/ml), streptomycin (0.1 mg/ml) and 10% (v/v) FBS at 37 °C and 5% CO_2_. Cell death was analyzed by PI (100 ng/ml), Sytox (5 nM) or Hoechst (1 mg/ml) staining coupled with microscopy or flow cytometry.

**Measurement of lipid hydroperoxides**

Lipid hydroperoxides was analyzed by flow cytometry: Cells were seeded at a density of 3.5 × 10^5^ per well in a 6-well dish and grown overnight in DMEM. 5 μM BODIPY C11 (Thermo Fisher, Cat# D3861) was added into cell culture medium and incubated for 30 min after indicated treatment. Excess BODIPY C11 was then removed by washing the cells with PBS twice. Labeled cells were trypsinized and resuspended in PBS plus 2% FBS. Oxidation of BODIPY C11 resulted in a shift of the fluorescence emission peak from 590 nm to 510 nm proportional to lipid hydroperoxides generation and was analyzed using a flow cytometer.

Lipid hydroperoxides imaging: Cells were seeded at a density of 2.5x10^5^ per well on coverslips placed in a 6-well dish and grown overnight in DMEM. Cells were treated as indicated. Coverslips were washed in HBSS and incubated in HBSS containing 2 mM BODIPY 581/591 C11 (Invitrogen) for 20 minutes. Coverslips were then inverted onto microscope slides. Slides were imaged using a ZEISS LSM 880 with fast Airyscan and images were processed in Photoshop

**Generation of stable cells.**

LKB1, ACC1 and FAS knockout cells were generated using CRISPR technology.

pLx-gRNA (Addgene#50662) expression plasmid encoding guide RNA was generated and co-transfected with Streptococcus pyogenes Cas9 expression plasmid and transfected into cells using Lipofectamine 2000 (Life Technologies). 24 h after the transfection, the cells were trypsinized and about 200 cells were seeded into a 10-cm plate. After cell clones were formed and expanded, western blot was performed to screening for knockout clones. The following guide RNA sequences were used:

Human LKB1 gRNA1 CAGGTGTCGTCCGCCGCGAACGG

Human LKB1 gRNA2 CAGCCGCCCGAGATTGCCAACGG

Human LKB1 gRNA3 CCACCGCATCGACTCCACCGAGG

Mouse LKB1 gRNA CCTCGGTGGAGTCGATGCGGTGG

Mouse ACC1 gRNA1 CAGAATTTGTTACTCGTTTTGGG

Mouse ACC1 gRNA2 AATGCATGCGATCTATC CGTCGG

Mouse ACC1 gRNA3 ACATTTCATAAGACCACCGACGG

Mouse FAS gRNA AACGAACACTGGCGTCTGGGTGG

MISSION lentiviral shRNA clones targeting human LKB1, AMPK and non-targeting control construct were purchased from Sigma-Aldrich. The clones ID for the shRNA targeting human LKB1-sh1: TRCN00000000409; AMPK-shRNA: TRCN00000000858. The sequence of mouse ACC1 shRNAs: #1 CCCAGCAGTATTTGAACACAT; #2 CCTGTGTGTTTGAGAAGGAAA. pCIP-AMPKa1_WT (Addgene Plasmid #79010) and pCIP-AMPKa1_KD (Addgene plasmid # 79011) were gifts from Reuben Shaw. pBABE-FLAG-LKB1 (Addgene plasmid # 8592) and pBABE-FLAG-KD LKB1 （Addgene plasmid # 8593）were gifts from Lewis Cantley.

Retrovirus or lentivirus was packaged in 293T cells and used to infect target cells which were then selected with puromycin for at least 3 days prior to use in experiments.

**NAD(P)H levels and NAD(P)H/NAD(P)^+^ Ratio Measurement**

MEF cells (1 × 10^6^ cells/sample) treated as indicated, were collected and intracellular NAD(P)H levels and NAD(P)H/NAD(P)^+^ Ratio were determined by using NAD^+^/NADH assay kit (S0175, Beyotime) or NADPH/NADP^+^ assay kit (S0179, Beyotime) according to the manufacturer’s instructions.

**Glutathione peroxidase assay**

MEF cells (2 × 10^6^ cells/sample) were collected and lysed in cold assay buffer and centrifuged at 10,000×g for 15 min at 4ºC and GPx activities was determined by Glutathione Peroxidase Assay Kit (ab102530, Abcam) according to the manufacturer's instructions.

**Mitochondrial Metabolic Potential Assay**

Cells were seeded into the XF96 microplates (4ⅹ10^4^ cells per well, Seahorse Bioscience) and incubated overnight at 37 °C. After indicated treatment, mitochondrial metabolic potential was measured by using Seahorse Cell Energy Phenotype Test Kit (Agilent Technologies) with Seahorse XFp Extracellular Flux Analyzer (Agilent Technologies) according to the manufacturer’s instructions. OCR (nmoles/min) were analyzed using the provided XFe Wave software (Seahorse Bioscience).

**Statistical analysis**

All statistical analyses were performed using Prism 5.0c GraphPad Software. *P* values were calculated with unpaired Student’s *t*-test. Data are presented as mean ± SD from 3 independent experiments. *P* <0.05 was set as the threshold for significance (**P* <0.05, ***P* <0.01, ****P<0.001*)
